# Herbal extracts in the treatment of Diabetic Foot Syndrome

**DOI:** 10.5195/cajgh.2013.86

**Published:** 2014-01-24

**Authors:** Tatyana Kustova, Tatyana Karpenyuk, Alla Goncharova, Leonid Mamonov, Samir Ross

**Affiliations:** 1 Department of Biology and Biotechnology, Al-Farabi Kazakh National University, Almaty, Kazakhstan; 2 Institute of Plant Biology and Biotechnology; 3 School of Pharmacy, University of Missisippi, Oxford, Mississippi

**Keywords:** diabetes, diabetic food syndrome, herbal extracts, Kazakhstan

## Abstract

**Introduction:**

One of the most serious complications of diabetes is the formation of Diabetic Foot Syndrome. Herbal extracts that combine high antioxidant and antimicrobial properties can be used to treat the resulting neuropathy.

The aim of this study was to determine antimicrobial and antioxidant activities of crude extracts isolated from plants growing in Kazakhstan, which could be used to develop products for treatment of Diabetic Foot Syndrome.

**Method:**

Different solvents, including dichloromethane and ethanol, were used to prepare plant extracts. The crude extracts from the plants were tested for antimicrobial activity using a modified version of the CLSI/NCCLS methods. All organisms were obtained from American Type Culture Collection. These included the fungi *Candida glabrata* ATTC 90030, the bacteria *Staphylococcus aureus* ATCC 29213, and *Methicillin-resistant S. aureus* ATCC 43300. The 2,2-diphеnyl-1-picrylhydrazyl (DPPH) assay, 2,2-azinobis (3-ethylbenzothiazoline-6-sulfonic acid) diammonium salt (ABTS) radical scavenging assay were used to analyzed the antioxidant capacity.

**Results:**

The results clearly indicate that antibacterial and antifungal activities vary with plant species. Dichloromethane extracts produced favorable results in all assays. *Epilobium hirsutum, Rhodiola quadrifida, Rumex confertus* showed antifungal activity against *Candida glabrata* in all extracts where IC_50_ less than 3 μg/ml. *Rumex confertus, Glycyrrhiza Uralensis and Vexibia alopecuroides* showed anti-fungal activity against *Staphylococcus aureus* (IC_50_ =10.80 μg/ml), (IC_50_ =11.10 μg/ml), (IC_50_ =3.05 μg/ml) and *Methicillin-resistant S. aureus* (IC_50_ =16.20 μg/ml), (IC_50_ =11.00 μg/ml), (IC_50_ =2.90 μg/ml) respectively. In spite of this, *Vexibia alopecuroides* extract showed no antioxidant activity. The other extracts showed a dose dependent ABTS scavenging activity. IC_50_ values were for the following: 6.6 μg/ml *Epilobium hirsutum;* 4.5 μg/ml *Rumex confertus;* 3.8 μg/ml *Rhodiola quadrifida,* 5.7 μg/ml *Glycyrrhiza Uralensis.* Extracts of *Epilobium hirsutum* and *Rumex confertus* had high antioxidant activity greater than 85% inhibition of DPPH (P ≤ 0.05).

**Conclusion:**

The demonstrated antimicrobial and antioxidant activities showed evidence supporting the use of herbal extracts to treat Diabetic Foot Syndrome.

